# Inhibition of methane and natural gas hydrate formation by altering the structure of water with amino acids

**DOI:** 10.1038/srep31582

**Published:** 2016-08-16

**Authors:** Jeong-Hoon Sa, Gye-Hoon Kwak, Kunwoo Han, Docheon Ahn, Seong Jun Cho, Ju Dong Lee, Kun-Hong Lee

**Affiliations:** 1 Department of Chemical Engineering, Pohang University of Science & Technology, 77 Cheongam-Ro, Nam-Gu, Pohang, Gyeongbuk 790-784, Korea; 2 CO_2_ Project Team, Research Institute of Industrial Science & Technology, 67 Cheongam-Ro, Nam-Gu, Pohang, Gyeongbuk 790-600, Korea; 3 Beamline Division, Pohang Accelerator Laboratory, 80 Jigok-Ro 127Beon-Gil, Nam-Gu, Pohang, Gyeongbuk 790-834, Korea; 4 Offshore Plant Resources R&D Center, Korea Institute of Industrial Technology, 30 Gwahaksandan 1-Ro 60Beon-Gil, Gangseo-Gu, Busan 618-230, Korea

## Abstract

Natural gas hydrates are solid hydrogen-bonded water crystals containing small molecular gases. The amount of natural gas stored as hydrates in permafrost and ocean sediments is twice that of all other fossil fuels combined. However, hydrate blockages also hinder oil/gas pipeline transportation, and, despite their huge potential as energy sources, our insufficient understanding of hydrates has limited their extraction. Here, we report how the presence of amino acids in water induces changes in its structure and thus interrupts the formation of methane and natural gas hydrates. The perturbation of the structure of water by amino acids and the resulting selective inhibition of hydrate cage formation were observed directly. A strong correlation was found between the inhibition efficiencies of amino acids and their physicochemical properties, which demonstrates the importance of their direct interactions with water and the resulting dissolution environment. The inhibition of methane and natural gas hydrate formation by amino acids has the potential to be highly beneficial in practical applications such as hydrate exploitation, oil/gas transportation, and flow assurance. Further, the interactions between amino acids and water are essential to the equilibria and dynamics of many physical, chemical, biological, and environmental processes.

As the readily accessible fossil fuel resources have become depleted, unconventional resources, such as shale gas/oil, tight gas, and coal-bed methane (CH_4_) have become more important. A substantial amount of natural gas (NG) is stored in gas hydrates, which are solid crystalline materials^1^ that physically resemble ice^2^ and contain the hydrocarbons in hydrogen-bonded water cages. Huge deposits of this energy source are found in permafrost and ocean sediments^3^,^4^, but the low commercial viability of its extraction, its geological implications, and the risks of exacerbating climate change have limited their exploitation^5^. Hydrates also hinder oil and gas transportation through pipelines, i.e., they create problems for flow assurance^6^,^7^. The occurrence of hydrate blockages in pipelines leads to shutdown and repair, so failures in hydrate management can lead to considerable financial losses and severe environmental damage.

The injection of additives is in principle a simple method for the control of hydrate formation^8^. Thermodynamic hydrate inhibitors (THIs) like alcohols and glycols permanently inhibit the formation of hydrates by shifting their phase equilibria to lower temperatures and higher pressures, but this approach requires the injection of vast amounts and supplementary facilities to deliver and recover the THIs. The use of kinetic hydrate inhibitors (KHIs), which delay hydrate nucleation and growth at low doses, is preferable for both economic and environmental reasons. However, it remains difficult to predict the kinetics of hydrate formation^9^, especially in the presence of KHIs, as it is a very complex and dynamic process. There have been numerous attempts to identify possible additives through molecular design and the testing of potential candidates^10^,^11^,^12^,^13^,^14^,^15^,^16^. More recently, the risk of environmental contamination has led to efforts to develop environmentally friendly additives. The amine (–NH_2_) and carboxylic acid (–COOH) groups of amino acids readily form hydrogen bonds with water molecules and the spontaneous formation of zwitterions enables electrostatic interactions, so their use as a new class of environmentally friendly additives has been proposed^17^,^18^. A systematic comparison of the inhibition efficiencies of amino acids is required. However, previous investigations into hydrate inhibition by amino acids have been limited to CO_2_^17^,^18^,^19^, ethane^20^, and tetrahydrofuran^21^ hydrates, although CH_4_ and NG hydrates are more important for flow assurance.

In this study, we examined the inhibition by amino acids of CH_4_ and NG hydrate formation. The alterations induced by amino acids in the structure of liquid water were found to interrupt the formation of particular hydrate cages and to affect the cage occupation characteristics of CH_4_ and NG hydrates. Thus amino acids have significant potential for industrial applications that require the inhibition of CH_4_ and NG hydrate formation such as the exploitation of hydrates, oil/gas pipeline transportation, and flow assurance. Further, the environmental friendliness of amino acids means that they can be used in areas with severe contamination risks.

## Results and Discussion

### Crystal structure and cage occupation behavior

**Figure 1:**
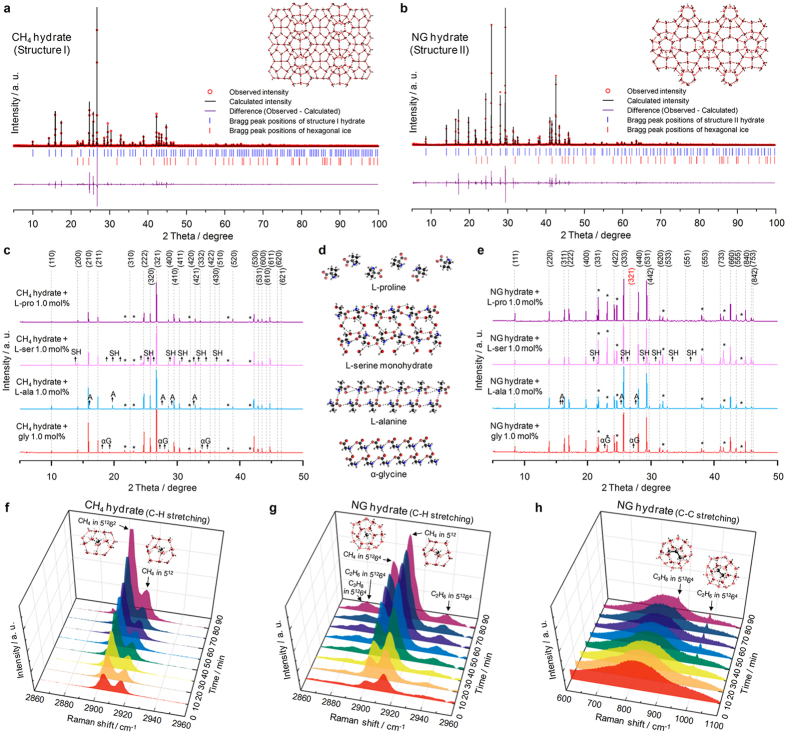
Crystal structure identification and hydrate cage occupation characteristics. (**a,b**) Synchrotron PXRD patterns of CH_4_ (**a**) and NG (**b**) hydrates at 80 K. (**c**) PXRD patterns of CH_4_ hydrates in the presence of the amino acids at 80 K. The peak positions for the structure I hydrate are indicated by the black Miller indices. The diffraction peaks for hexagonal ice (*), α-glycine (αG), L-alanine (A), and L-serine monohydrate (SH) crystals are labeled. (**d**) Structural features of the molecular arrangements of the amino acids as revealed by PXRD. The addition of the amino acids has almost no influence on the hydrate crystal structures. The amino acids glycine, L-alanine, and L-serine self-crystallize when they are excluded from the hydrate phase, but L-proline is soluble in water. (**e**) PXRD patterns of NG hydrates in the presence of the amino acids at 80 K. The peak positions for the structure II hydrate are indicated by the black Miller indices. The additional diffraction peak for the structure I hydrate is shown as a red Miller index. (**f**) *In situ* Raman spectra of CH_4_ hydrates at 274.15 K. CH_4_ occupies both the small 5^12^ and large 5^12^6^2^ cages of structure I. (**g,h)** *In situ* Raman spectra of the NG hydrate at 274.15 K for the C-H (**g**) and C-C (**h**) stretching regions. CH_4_ occupies both small 5^12^ and large 5^12^6^4^ cages of structure II, whereas C_2_H_6_ and C_3_H_8_ only occupy large 5^12^6^4^ cages.

The crystal structures of CH_4_ and NG (93% CH_4_, 5% C_2_H_6_, 2% C_3_H_8_) hydrates were characterized with synchrotron powder X-ray diffraction (PXRD). CH_4_ forms structure I hydrates consisting of 5^12^ and 5^12^6^2^ cages (Fig. 1a). NG forms structure II hydrates consisting of 5^12^ and 5^12^6^4^ cages (Fig. 1b). The presence of 5% C_2_H_6_ and 2% C_3_H_8_ alters hydrate crystal structures^22^,^23^,^24^. Although the addition of amino acids to CH_4_ hydrates does not alter the crystal structure, as is the case for the CO_2_ hydrate system^18^,^25^, diffraction peaks for hexagonal ice are evident (Fig. 1c), which indicates that the conversion of water to hydrates has been interrupted and that the liquid water freezes during the liquid N_2_ quenching^18^,^25^. However, the lattice parameters of CH_4_ hydrates containing amino acids do not have any correlation with the size or hydrophobicity values of amino acids (Supplementary Table S2), inconsistent with the CO_2_ hydrate system^25^. This result implies that the addition of amino acids affects diverse aspects of CH_4_ hydrate formation as well as the crystal structures. Additional diffraction peaks due to the crystals of the amino acids are observed as the concentration of the amino acids increases, i.e. thermodynamically stable amino acid phases form, as observed in the CO_2_ hydrate system. Glycine and L-alanine self-crystallize, and L-serine forms monohydrates due to its high affinity with water (Fig. 1d). L-Proline does not crystallize as it is highly soluble. Similar results were obtained for the NG hydrates (Fig. 1e); the only difference is the presence of a weak diffraction peak for the structure I hydrate, which is consistent with previous reports^22^,^23^,^24^.

The hydrate cage occupation characteristics were determined by obtaining *in situ* Raman spectra. CH_4_ occupies both the 5^12^ and 5^12^6^2^ cages of the structure I hydrate and the 5^12^6^2^ peak is more prominent (Fig. 1f); the stoichiometric ratio of these cages is 1:3^26^. The respective *I*_*L*_*/I*_*S*_ value of the CH_4_ hydrate is 3.02, though this value is not identical to the cage filling ratio^27^. In contrast, the NG structure II hydrate contains more 5^12^ than 5^12^6^4^ cages (Fig. 1g,h). The C-H and C-C peaks indicate that C_2_H_6_ and C_3_H_8_ only occupy 5^12^6^4^ cages because they are much larger than CH_4_^22^. Heavier hydrocarbons have more influence on the hydrate crystal structure. This feature is typical of naturally occurring hydrates, including structure II and structure H hydrates^28^.

### Phase equilibria and thermodynamic inhibition

**Figure 2:**
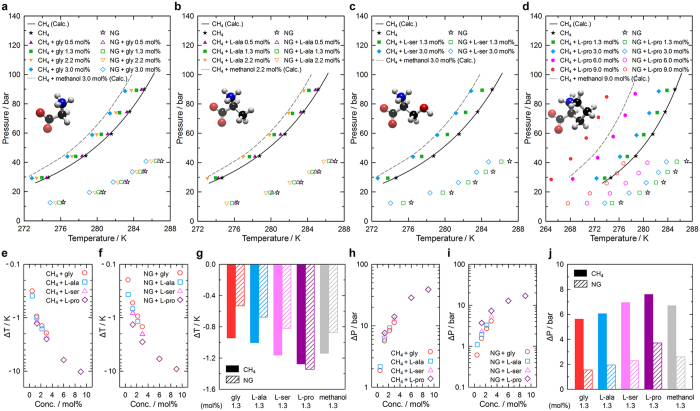
Hydrate formation conditions and thermodynamic inhibition by amino acids. (**a–d)** Phase equilibria of CH_4_ and NG hydrates in the presence of glycine (**a**), L-alanine (**b**), L-serine (**c**), and L-proline (**d**). Phase equilibrium conditions of pure CH_4_ and methanol added systems calculated by using CSMGem^50^ are added for comparison. The amino acids shift the hydrate formation conditions to lower temperature and higher pressure regions. (**e,f**) The extents of the shifts in the phase equilibrium temperatures obtained by adding amino acids to CH_4_ (**e**) and NG (**f**) hydrates. The extents of the temperature shifts generally increase with concentration, but distinct differences between the amino acids were found. (**g)** Quantitative comparison of the THI efficiencies of the amino acids at 1.3 mol% according to the temperature shifts: L-proline > L-serine > L-alanine > glycine. (**h,i)** The extents of the shifts in the phase equilibrium pressure upon the addition of the amino acids to CH_4_ (**h**) and NG (**i**) hydrates. (**j)** Quantitative comparison of the THI efficiencies of the amino acids at 1.3 mol% according to the pressure shifts. The trend in the pressure shifts is very similar to that in the temperature shifts.

The thermodynamic inhibition effects of the amino acids on CH_4_ and NG hydrate formation were investigated by characterizing the hydrate formation conditions. The CH_4_ hydrate phase equilibria appear in the 30 to 90 bar region in the temperature range 274.65 to 285.15 K (Fig. 2a). NG hydrates form at much lower pressures because C_2_H_6_ and C_3_H_8_ readily occupy 5^12^6^4^ cages. This issue is critical for pipeline flow assurance because the presence of heavier hydrocarbons leads to serious hydrate plugging risks. The presence of glycine shifts the formation conditions of both CH_4_ and NG hydrates to lower temperature and higher pressure regions, as is the case for the CO_2_ hydrate system^17^, and the extents of these shifts increase with the glycine concentration (Fig. 2a). The other amino acids have similar effects (Fig. 2b–d). In particular, L-serine and L-proline were first tested as THIs in the present study. The amino acids tested here have significant potential as THIs regardless of the hydrate crystal structure, and thus are expected to be useful in practical applications. In addition, their environment friendliness provides lower environmental risks.

The extents of the temperature shifts upon the addition of the amino acids were calculated (see the Supplementary Information) for quantitative comparison (Fig. 2e,f). There are slight differences between the temperature shifts of the amino acids: L-proline > L-serine > L-alanine > glycine (Fig. 2g). The trend in the pressure shifts is identical (Fig. 2h–j). Glycine contains −NH_2_ and −COOH groups, so forms zwitterions in water. The resulting hydrogen bonds and electrostatic interactions lower the activity coefficient of water, and thus lead to thermodynamic inhibition^1^. The additional methyl (−CH_3_) group of L-alanine and the further hydroxyl (−OH) group of L-serine are expected to result in stronger interactions of these amino acids with water. L-Proline, in particular, exhibits an outstanding inhibition efficiency, and the introduction of 9.0 mol% L-proline even shifts the formation conditions of NG hydrates to those of pure CH_4_ hydrates. This powerful effect originates in its structural peculiarity: even though L-proline possesses both hydrophobic and hydrophilic moieties, the intermolecular hydrophobic stacking of its pyrrolidine rings greatly enhances its hydrophilicity^29^. This ring structure also significantly reduces the loss of internal mobility when dissolved in water^30^. L-Proline facilitates the osmoprotection of plants against drought stress and the cold hardening of living organisms^31^.

### Nucleation/growth kinetics and kinetic inhibition

**Figure 3:**
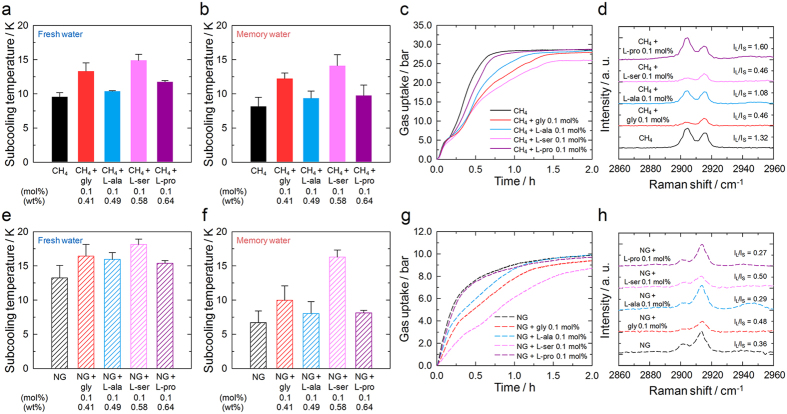
Hydrate formation kinetics and kinetic inhibition by amino acids. (**a,b**) Heterogeneous nucleation kinetics of CH_4_ hydrates in the presence of 0.1 mol% amino acids in fresh (**a**) and memory (**b**) water. An increase in the subcooling temperature indicates enhanced KHI performance. (**c)** Growth kinetics of CH_4_ hydrates in the presence of 0.1 mol% amino acids. Glycine and L-serine delay CH_4_ hydrate nucleation and growth in both fresh and memory water, whereas L-alanine and L-proline have negligible influence. (**d)** Raman spectra of CH_4_ hydrates during the early stages of growth. *I*_*L*_*/I*_*S*_ is the ratio of the peak intensities of the large (5^12^6^2^ or 5^12^6^4^) and small (5^12^) hydrate cages. Glycine and L-serine obviously retard the occupation of 5^12^6^2^ cages by CH_4_, and thus reduce the growth rate. (**e,f)** Heterogeneous nucleation kinetics of NG hydrates in the presence of 0.1 mol% amino acids in fresh (**e**) and memory (**f**) water. (**g)** Growth kinetics of NG hydrates in the presence of 0.1 mol% amino acids. The trend in the KHI performances of the amino acids for NG hydrates is very similar to that for CH_4_ hydrates. (**h)** Raman spectra of NG hydrates during the early stages of growth. Glycine and L-serine significantly reduce the intensities of the Raman peaks for CH_4_ in both 5^12^ and 5^12^6^4^ cages.

The amino acids also affect hydrate formation kinetics. Glycine and L-serine delay CH_4_ hydrate nucleation whereas L-alanine and L-proline do not (Fig. 3a,b). L-Alanine was found to be effective in CO_2_ hydrate inhibition in a previous study^18^, but has no influence on CH_4_ hydrate formation. Although it is more difficult to inhibit hydrate nucleation in memory water because it retains thermal history^1^, glycine and L-serine are promising KHIs in both fresh and memory water. A similar trend was found in the growth kinetics (Fig. 3c). In the presence of KHIs, the rate of CH_4_ hydrate formation can be lowered, especially at the intermediate stage^32^,^33^. One unexpected result is the poor KHI efficiency of L-proline, which is the best THI of the tested amino acids. According to a previous study, the hydrophobicity of amino acids is critical to their THI efficiencies for CO_2_ hydrates^17^. The THI efficiencies of the amino acids for the CH_4_ hydrates are in general agreement with that finding, with the exception of L-proline; its high inhibition efficiency is attributed to its structural peculiarity, as discussed above. It is clear that the inhibition mechanisms of THIs and KHIs are very different. Enhancing the hydrophilicity of KHIs does not guarantee superior inhibition efficiency.

The cage occupation characteristics of CH_4_ in the early growth stages are intriguing (Fig. 3d). In the presence of L-alanine or L-proline, the 5^12^6^2^ peaks are prominent, as for a typical structure I hydrate (see the Supplementary Information). However, the presence of glycine or L-serine reduces the intensities of the 5^12^6^2^ peaks considerably in the initial growth stage, which indicates that these amino acids render the formation of hydrate cages and their occupation by CH_4_ much more difficult. The higher KHI efficiencies of these amino acids might arise as a consequence of this phenomenon. After hydrate formation has finished, the *I*_*L*_*/I*_*S*_ values of the systems containing glycine or L-serine are lower, as a result of the inhibition of 5^12^6^2^ cage occupation (Fig. S2). Although there have been previous simulations of the formation and inhibition of hydrates^11^,^34^,^35^,^36^,^37^,^38^, our direct experimental observation of hydrate formation has demonstrated the selective inhibition of hydrate cages and revealed the correlation of this inhibition with solute properties.

The amino acids have similar effects on the NG hydrates; L-serine was found to exhibit a much higher KHI efficiency (Fig. 3e–g). In the early growth stage, the presence of glycine or L-serine reduces the intensities of the 5^12^ and 5^12^6^4^ peaks, but according to their respective *I*_*L*_*/I*_*S*_ values the 5^12^ peak is even more affected (Fig. 3h). However, this observation is difficult to interpret because C_2_H_6_ and C_3_H_8_ compete with CH_4_ to occupy 5^12^6^4^ cages. The similarity of the trends in the KHI efficiencies of the amino acids for CH_4_ and NG hydrates is truly remarkable given that the arrangements of water molecules in hydrate cages and their modes of stacking are entirely different for structure I and II hydrates^1^. Thus the important considerations for hydrate inhibition are the dissolution environment of the additive and the physicochemical phenomena of the hydrate formation process.

### Perturbation of the structure of water

**Figure 4:**
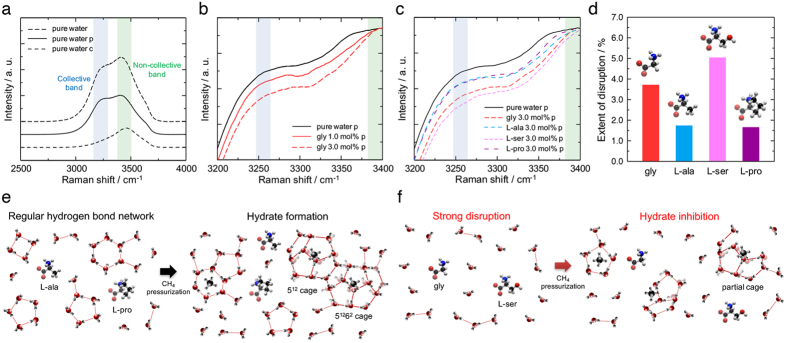
Perturbation of the structure of liquid water. (**a)** Polarized Raman spectra of liquid water for parallel (p) and cross (c) polarizations. The low frequency band originates from the collective in-phase stretching motion of hydrogen-bonded water molecules. (**b,c)** Collective bands for the parallel polarization in the polarized Raman spectra of aqueous glycine (**b**) and amino acid (**c**) solutions. Each amino acid has a distinctive influence on the collective motion of water molecules. (**d)** Quantitative comparison of the extents of water structure disruption by the amino acids. (**e,f)** Hypothetical representations of the hydrate formation and inhibition phenomena. In the presence of L-alanine and L-proline (**e**), the water molecules are connected by intermolecular hydrogen bonds, and thus hydrate formation proceeds. In contrast, glycine and L-serine (**f**) strongly disrupt the water hydrogen bond network, so the arrangement of water molecules to form hydrate cages, especially large cages, is considerably retarded.

The structural characteristics of liquid water were examined by obtaining polarized Raman spectra. The presence of a solute in water causes changes in the hydrogen bond network that depend on its physical properties^39^,^40^,^41^,^42^. The low frequency band near 3250 cm^−1^ only arises for parallel polarization (Fig. 4a). This highly polarized collective band can be used as an indicator of the strengthening or disruption of hydrogen bond networks^43^,^44^. The presence of glycine only slightly reduces the intensity of this collective band, as is consistent with previous reports^45^,^46^, but the extent of this reduction increases with the glycine concentration, which indicates increased disruption of the water structure (Fig. 4b). According to our calculations of the extent of water structure disruption (see the Supplementary Information) from the polarized Raman spectra (Fig. 4c), glycine and L-serine strongly disrupt the structure of water, whereas L-alanine and L-proline have only weak effects (Fig. 4d).

Interestingly, the trend in the polarized Raman spectra is analogous to that found in the hydrate kinetics. Thus, it can be hypothesized that the introduction of CH_4_ leads to the formation of 5^12^ and 5^12^6^2^ cages as in regular hydrate formation, because L-alanine and L-proline have little influence on the structure of water (Fig. 4e). However, the disruptions of the hydrogen bond network are incompatible with the formation of hydrate cages^36^ because of the increased barrier to hydrate nucleation. This selective inhibition of hydrate cages significantly delays the growth of hydrates (Fig. 4f). Although the influence of water structure perturbation on hydrate inhibition has previously been proposed^18^,^46^, the selective inhibition of the formation of particular hydrate cages was demonstrated for the first time in the present study. Our examinations of tetrahydrofuran hydrate crystal morphology (see the Supplementary Information) also indicate that a previous hypothesis that the inhibition mechanism is based on the adsorption of KHIs onto hydrate surfaces^34^ is not applicable to the hydrate inhibition provided by amino acids, although the growth mechanism of tetrahydrofuran hydrates is expected to be somewhat different. Our findings demonstrate that the perturbation of the structure of liquid water by amino acids can affect the formation, inhibition, and occupation of hydrate cages, and that these phenomena determine the hydrate inhibition characteristics.

## Conclusion

In this study, we investigated the inhibition of CH_4_ and NG hydrate formation by amino acids and the associated physicochemical phenomena. L-Proline was found to be an outstanding THI due to its extremely hydrophilic nature. This result demonstrates the importance of the direct interaction of the additive with water. L-Serine is the best KHI of the amino acids in terms of inhibition of both hydrate nucleation and growth. To explain the difference between the THI and KHI efficiencies, the perturbations of the structure of liquid water by the amino acids were directly observed. The disruption of the water hydrogen bond network by glycine and L-serine significantly delay hydrate formation, which means that the dissolution environment of the additive and its influence on the physicochemical phenomena of hydrate formation are crucial. An intimate connection was found between these perturbations and the selective inhibition of hydrate cages, which establishes a new perspective on molecular behaviors in the aqueous phase during hydrate formation and inhibition. The environmentally friendly nature of amino acids and their potential in CH_4_ and NG hydrate inhibition are highly advantageous to practical applications in oil and gas pipeline transportation, especially in areas with severe contamination risks. Further, this new understanding of the interactions between amino acids and water and of the resulting effects on its structure will be useful for research into the equilibria and dynamics of a variety of physicochemical and environmental processes.

## Methods

### PXRD

The hydrate samples for PXRD characterization were synthesized by using a high pressure cell system^18^, the SUS 315 cell, which has a volume of 250 cm^3^ and can withstand pressures up to 200 bar. This high pressure cell was immersed in an ethanol bath in order to control its temperature. The cell temperature and pressure were measured with a K-type thermocouple (±0.1 K) and a WIKA A-10 pressure transmitter (±0.5%) respectively. Each aqueous solution placed in the cell was agitated with an impeller coupled to a magnetic drive. Each aqueous amino acid solution was prepared by mixing the amino acid with 40 g of water, and then adding it to the high pressure cell. The cell was pressurized with up to 90 bar of CH_4_ or 45 bar of NG at 287.95 K. The cell temperature was held constant for 1 h with agitation at 450 rpm, and then cooled to 273.45 K without agitation. When the temperature reached 273.45 K, agitation was resumed to induce hydrate formation. Each hydrate sample was synthesized for 15 h, and ground into a fine powder in a liquid N_2_ environment. The synchrotron PXRD characterizations were performed at the 9B high resolution powder diffraction beamline of the Pohang Accelerator Laboratory. The incident X-rays were monochromatized to a wavelength of 1.46470 or 1.46390 Å by using a DCM Si(111) crystal. Each hydrate sample was placed on a Cu plate holder, and a cryostat was used to control the sample temperature in the range 80.0 to 200.0 K. Each step scan was carried out with a fixed time of 1.0 s and a step size in 2θ of 0.02° from 5.00° to 125.50° with a 0.50° overlap. The PXRD patterns were analyzed with two-phase (hydrate + hexagonal ice) or three-phase (hydrate + hexagonal ice + amino acid) profile matching^25^ by using the FullProf program^47^.

### *In situ* Raman spectroscopy

The *in situ* Raman measurements were performed at the Korea Institute of Industrial Technology. The details of the *in situ* Raman spectroscopy system have previously been documented^48^,^49^. The SUS 315 high pressure cell and SUS 315 supply vessel have volumes of 333 mL and 1848.3 mL respectively. The cell was immersed in a water bath filled with an aqueous ethanol solution to control the system temperature. The cell temperature and pressure were monitored with an OMEGA Pt100 RTD and a GE UNIK 5000 pressure transmitter respectively. Each aqueous amino acid solution was prepared by mixing the amino acid with 100 g of water, and then added to the cell. The cell was pressurized with up to 70 bar of CH_4_ or 45 bar of NG at 274.15 K without agitation, and then agitation at 300 rpm was commenced. Once hydrate formation had commenced, CH_4_ or NG was supplied from the supply vessel to maintain a constant pressure. Each experiment was carried out for at least 80 min. The Raman spectrometer was produced by Lambda Ray Co. (Korea) and contains a mirror-type probe coupled with a CCD detector that can withstand pressures up to 150 bar. A Nd:YAG laser with a wavelength of 532 nm and a power of 100 mW was used. The scans for the Raman spectra were performed in the range 2573.4 to 4580.9 cm^−1^ with a resolution of 0.8 cm^−1^ for the CH_4_ hydrates and in the range 475.5 to 3013.6 cm^−1^ with a resolution of 1.0 cm^−1^ for the NG hydrates.

### Phase equilibrium measurements

The hydrate phase equilibria were investigated with the high pressure cell system used for PXRD sample preparation. Our experimental procedure for the phase equilibrium measurements was as previously described^17^. An aqueous amino acid solution obtained by mixing one of the amino acids with 60 g of water was placed in the cell, and CH_4_ or NG was pressurized up to the desired pressure at 287.95 K. The cell was then cooled to initiate hydrate formation, which produces a dramatic pressure drop. After hydrate formation, the cell was heated at a rate of 0.1 K/h to dissociate the hydrates. Once the hydrates had dissociated completely, the phase equilibrium was identified by determining the intersection point of the cooling and heating curves on a pressure-temperature plot. In order to compare the THI performances of the amino acids, Δ*T* and Δ*P* were calculated with the following equations:


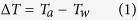



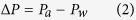


where *T*_*a*_ (or *P*_*a*_) is the phase equilibrium temperature (or pressure) of the system containing an aqueous amino acid solution at a given pressure (or temperature), and *T*_*w*_ (or *P*_*w*_) is the phase equilibrium temperature (or pressure) of a pure water system at a given pressure (or temperature). The regression equations obtained by fitting the phase equilibrium conditions were used. Lower Δ*T* values and higher Δ*P* values correspond to better THI performances.

### Kinetics measurements

The heterogeneous nucleation kinetics of these systems in fresh water were investigated with the constant cooling method, as described previously^18^,^46^. The high pressure cell was filled with an aqueous amino acid solution, and pressurized up to 90 bar of CH_4_ or 40 bar of NG at 288.95 K with agitation at 450 rpm, then the cell was cooled at a rate of 0.25 K/min. When hydrate nucleation occurs, the cell temperature suddenly increases because nucleation is exothermic. The subcooling temperature at the onset of hydrate nucleation, *T*_*sub*_, was calculated with the following equation:


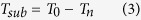


where *T*_*0*_ is the phase equilibrium temperature of a pure water system at the onset pressure of hydrate nucleation and *T*_*n*_ is the onset temperature of hydrate nucleation. Higher *T*_*sub*_ values correspond to better nucleation inhibition performances. The heterogeneous nucleation kinetics of these systems in memory water were investigated under constant cooling with the superheated hydrate method, as described previously^18^,^46^. The only difference between this experimental procedure and that described above was the use of memory water, which retains the thermal history of the hydrates. After the formation of hydrates through the constant cooling method, the cell was heated to 288.95 K without agitation. The hydrates dissociated until the cell pressure reached 89 bar of CH_4_ or 39.5 bar of NG. Agitation at 450 rpm was then applied for 30 min, and the procedures of the constant cooling method were repeated. The growth kinetics of the hydrates were measured with the isothermal method. Each high pressure cell containing an aqueous amino acid solution was pressurized up to 90 bar of CH_4_ or 40 bar of NG at 288.95 K with agitation at 450 rpm, and quickly cooled to 273.45 K without agitation. The agitation was then resumed to induce hydrate formation. The initiation of hydrate formation produces a dramatic increase in cell temperature, as in the above experiments. The rate of gas uptake during hydrate growth was measured for at least 10 h after the initiation of hydrate formation.

### Polarized Raman spectroscopy

The polarized Raman spectra of the aqueous amino acid solutions were obtained at the Raman Research Center. The details of the experimental system have previously been described^46^. The LabRam ARAMIS Raman spectrometer was produced by Horiba Jobin Yvon. An Ar-ion laser with a wavelength of 514 nm and a power of 2 mW was used. After loading a droplet of an aqueous amino acid solution (0.1 mL) into the spectrometer, the scan was performed in the range 2500 to 4000 cm^−1^ with a resolution of 2.0 cm^−1^. The extent of the perturbation of the structure of water by the amino acids was quantified with the parameter *C*, which is defined by the following equation:


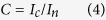


where *I*_*c*_ is the maximum intensity of the collective band centered at ~3250 cm^−1^ on the polarized Raman spectrum for the parallel polarization and *I*_*n*_ is that of the non-collective band centered at ~3400 cm^−1^. To compare the influences of the amino acids on the water hydrogen bond network, the extent of water structure disruption was obtained as follows:


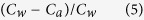


where *C*_*w*_ and *C*_*a*_ are the *C* values of pure water and the aqueous amino acid solution respectively.

## Additional Information

**How to cite this article**: Sa, J.-H. *et al*. Inhibition of methane and natural gas hydrate formation by altering the structure of water with amino acids. *Sci. Rep.* **6**, 31582; doi: 10.1038/srep31582 (2016).

## Supplementary Material

Supplementary Information
