# Supplementary material for: Inhibition of methane and natural gas hydrate formation by altering the structure of water with amino acids
Source: Sci Rep. 2016 Aug 16;6:31582. doi: 10.1038/srep31582 (PMC4985706; doi:10.1038/srep31582)
Supplement: Supplementary Information [file srep31582-s1.pdf]

Supplementary Information for:

Inhibition of methane and natural gas hydrate formation by altering the structure of water with amino acids

Jeong-Hoon Sa[1*], Gye-Hoon Kwak[1], Kunwoo Han[2], Docheon Ahn[3], Seong Jun Cho[4], Ju Dong Lee[4], and Kun-Hong Lee[1*]

[1]*Department of Chemical Engineering, Pohang University of Science & Technology, 77 Cheongam-Ro, Nam-Gu, Pohang, Gyeongbuk 790-784, Korea*

[2]*CO_2 Project Team, Research Institute of Industrial Science & Technology, 67 Cheongam-Ro, Nam-Gu, Pohang, Gyeongbuk 790-600, Korea*

[3]*Beamline Division, Pohang Accelerator Laboratory, 80 Jigok-Ro 127Beon-Gil, Nam-Gu, Pohang, Gyeongbuk 790-834, Korea*

[4]*Offshore Plant Resources R&D Center, Korea Institute of Industrial Technology, 30 Gwahaksandan 1-Ro 60Beon-Gil, Gangseo-Gu, Busan 618-230, Korea*

*Corresponding authors:
Dr. Jeong-Hoon Sa; Tel: 82-54-279-5305; Fax: 82-54-279-5949; e-mail: jumuri@postech.ac.kr
Prof. Kun-Hong Lee; Tel: 82-54-279-2271; Fax: 82-54-279-8298; e-mail: ce20047@postech.ac.kr

Present address of Dr. Jeong-Hoon Sa:
Chemical & Biological Engineering Department, Colorado School of Mines, 1500 Illinois Street, Golden, Colorado 80401, Unites States

Correspondence and requests for materials should be addressed to J.H.S. (e-mail: jumuri@postech.ac.kr) or K.H.L. (e-mail: ce20047@postech.ac.kr)

1. Materials and methods

Materials

Distilled water was prepared in the laboratory. Ultra-high purity CH_4 (99.999%) was supplied by KOREA NOBLE GAS Co. (Korea), and the NG mixture (CH_4 93.0%, C_2H_6 5.0%, C_3H_8 2.0%) was obtained from PS CHEM Co. (Korea). The chemicals used in this study, including glycine, L-alanine, L-serine, L-proline, polyvinyl pyrrolidone (PVP), and tetrahydrofuran (THF) were purchased from Sigma-Aldrich, USA. All chemicals were of reagent grade (≥99%) and used without further purification.

Crystal morphology observation

THF hydrate crystals were synthesized by using a glass pipette (Fig. S1). The experimental procedure was similar to that in previous reports[1,2]. A glass test tube was filled with an aqueous THF solution with a concentration of 5.56 mol%, and closed with a rubber stopper coupled with a glass pipette. The test tube was then immersed in an ethylene glycol bath to maintain its temperature at 276.15 K. This temperature is 1.4 K above the phase equilibrium temperature of THF hydrates under atmospheric conditions[3]. To initiate THF hydrate formation, a thin Cu wire cooled in liquid N_2 was inserted into the glass pipette. As the growth front reached the end of the glass pipette, a small THF hydrate crystal grew into the aqueous phase. This procedure was repeated with aqueous THF solutions containing either 0.1 mol% of each amino acid or 0.5 wt% PVP.

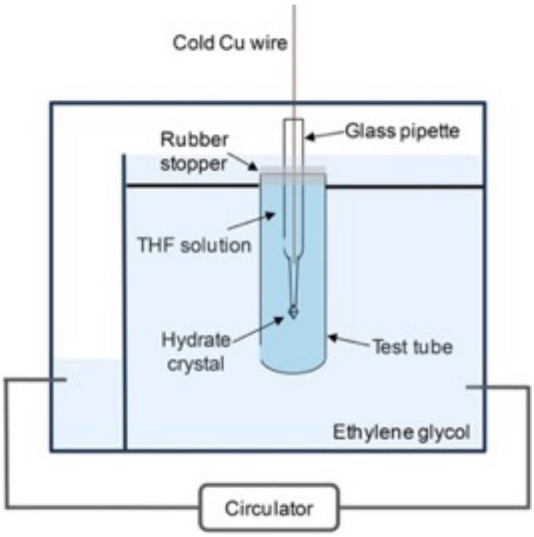

Figure S1. Experimental system for THF hydrate crystal morphology observation.

2. Model amino acids

Each amino acid has different characteristic physical and chemical properties, which strongly depend on its side chains. The properties of the amino acids tested in this study are summarized in Table S1.

Table S1. The physicochemical properties of the model amino acids.

Amino acid	Glycine (gly)	Alanine (ala)	Serine (ser)	Proline (pro)
Molecular structure	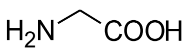	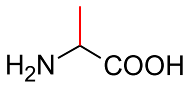	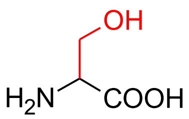	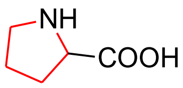
Side chain	-H	$-CH_3$	$-CH_2OH$	$-CH_2CH_2CH_2-$
Solubility in water at 273.15 K (mol%)[a]	3.32	2.51	3.00	16.63
Hydrophobicity[b]	1.8	-0.4	-0.8	-1.6
pK_{a1} (-COOH) at 273.15 K[c]	2.39	2.41	2.23	1.99
pK_{a2} ($-NH_2$) at 273.15 K[c]	10.43	10.32	9.80	11.29

[a]Solubility values were obtained from previous reports[4–6].

[b]Hydrophobicity values were obtained from a previous report[7].

[c]Acid dissociation constants at 273.15 K were calculated by using the Van't Hoff equation and literature values from previous reports[8–10].

3. Lattice parameter calculations

The lattice parameter values were calculated from the PXRD patterns by performing profile matching[11] with the FullProf program[12].

Table S2. Calculated lattice parameters of CH_4 hydrates in the presence of amino acids.

Temperature (K)	Lattice parameter (Å)				
	Pure water	gly 1.0 mol%	L-ala 1.0 mol%	L-ser 1.0 mol%	L-pro 1.0 mol%
80	11.848886 ± 0.000059	11.846941 ± 0.000087	11.850635 ± 0.000086	11.840753 ± 0.000044	11.847330 ± 0.000060
110	11.865589 ± 0.000059	-	-	-	-
140	11.875465 ± 0.000063	-	-	-	-
170	11.887923 ± 0.000167	-	-	-	-
200	11.907848 ± 0.000136	-	-	-	-

Table S3. Calculated lattice parameters of NG hydrates in the presence of amino acids.

Temperature (K)	Lattice parameter (Å)				
	Pure water	gly 1.0 mol%	L-ala 1.0 mol%	L-ser 1.0 mol%	L-pro 1.0 mol%
80	17.119015 ± 0.000104	17.114351 ± 0.000191	17.105139 ± 0.000194	17.118622 ± 0.000173	17.104891 ± 0.000145
110	17.133560 ± 0.000097	-	-	-	-
140	17.150503 ± 0.000109	-	-	-	-
170	17.170921 ± 0.000111	-	-	-	-
200	17.200790 ± 0.000563	-	-	-	-

4. Crystallographic information

Table S4. Crystallographic information for the amino acid crystals.

Amino acid	Crystal system	Space group	Temperature (K)	Cell dimension (Å, °)	Reference
α-glycine	Monoclinic	$P2_1/n$ (No. 14)	298	a = 5.1020(8), b = 11.971(2), c = 5.458(2) α = γ = 90, β = 111.42(3)	*Acta Cryst.* **11**, 654-663 (1958).
			120	a = 5.084(1), b = 11.820(2), c = 5.4579(9) α = γ = 90, β = 111.95(2)	*Acta Cryst.* **B36**, 3052-3059 (1980).
β-glycine	Monoclinic	$P2_1$ (No. 4)	298	a = 5.077(4), b = 6.267(6), c = 5.379(9) α = γ = 90, β = 113.12	*Acta Cryst.* **13**, 35-45 (1960).
γ-glycine	Trigonal	$P3_1$ (No. 144)	298	a = b= 7.037, c = 5.483 α = β = 90, γ = 120	*Acta Cryst.* **11**, 225-226 (1958).
			83	a = b= 6.975(2), c = 5.473(2) α = β = 90, γ = 120	*Acta Cryst.* **B36**, 115-120 (1980).
L-alanine	Orthorhombic	$P2_12_12_1$ (No. 19)	298	a = 6.032(1), b = 12.343(1), c = 5.784(1) α = β = γ = 90	*Acta Cryst.* **20**, 550-555 (1966).
			23	a = 5.928(1), b = 12.260(2), c = 5.794(1) α = β = γ = 90	*J. Phys. Chem.* **92**, 966-973 (1988).
L-serine	Orthorhombic	$P2_12_12_1$ (No. 19)	298	a = 8.599(5), b = 9.348(3), c = 5.618(2) α = β = γ = 90	*Acta Cryst.* **B30**, 2573-2578 (1974).
L-serine monohydrate	Orthorhombic	$P2_12_12_1$ (No. 19)	298	a = 9.365(7), b = 12.239(9), c = 4.835(7) α = β = γ = 90	*Acta Cryst.* **B29**, 876-884 (1973).
			97	a = 4.7987(4), b = 9.3063(9), c = 12.1460(11) α = β = γ = 90	*J. Phys. Chem. A* **114**, 9570-9578 (2010).
L-proline	Orthorhombic	$P2_12_12_1$ (No. 19)	298	a = 11.550, b = 9.020, c = 5.200 α = β = γ = 90	*Kristallografiya* **10**, 833-844 (1965).

5. In situ Raman spectra

The in situ Raman spectra of the CH_4 (Fig. S2) and NG hydrates (Fig. S3) in the presence of amino acids were recorded.

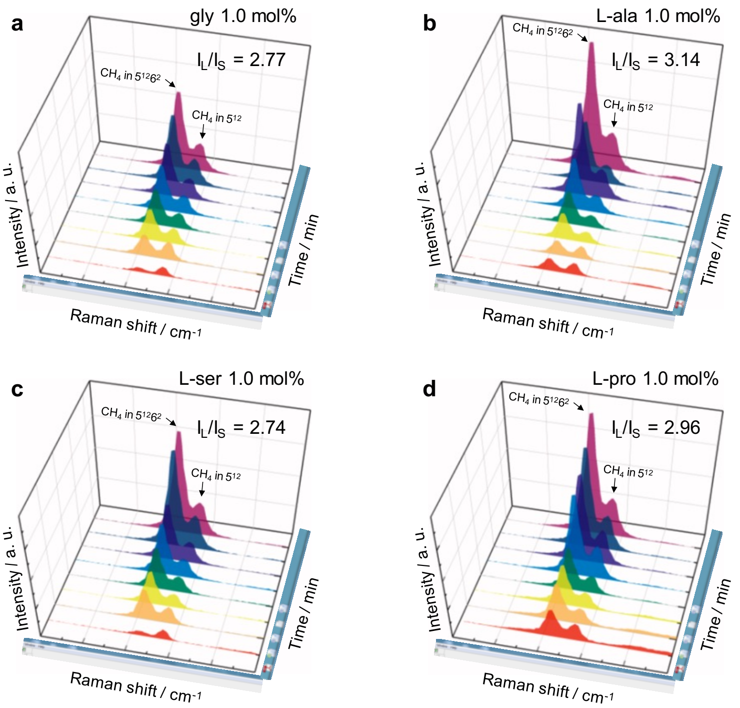

Figure S2. In situ Raman spectra of CH_4 hydrates at 274.15 K in the presence of (a) glycine, (b) L-alanine, (c) L-serine, and (d) L-proline for the C-H stretching region. I_L/I_S is the ratio of the peak intensities of the large and small hydrate cages.

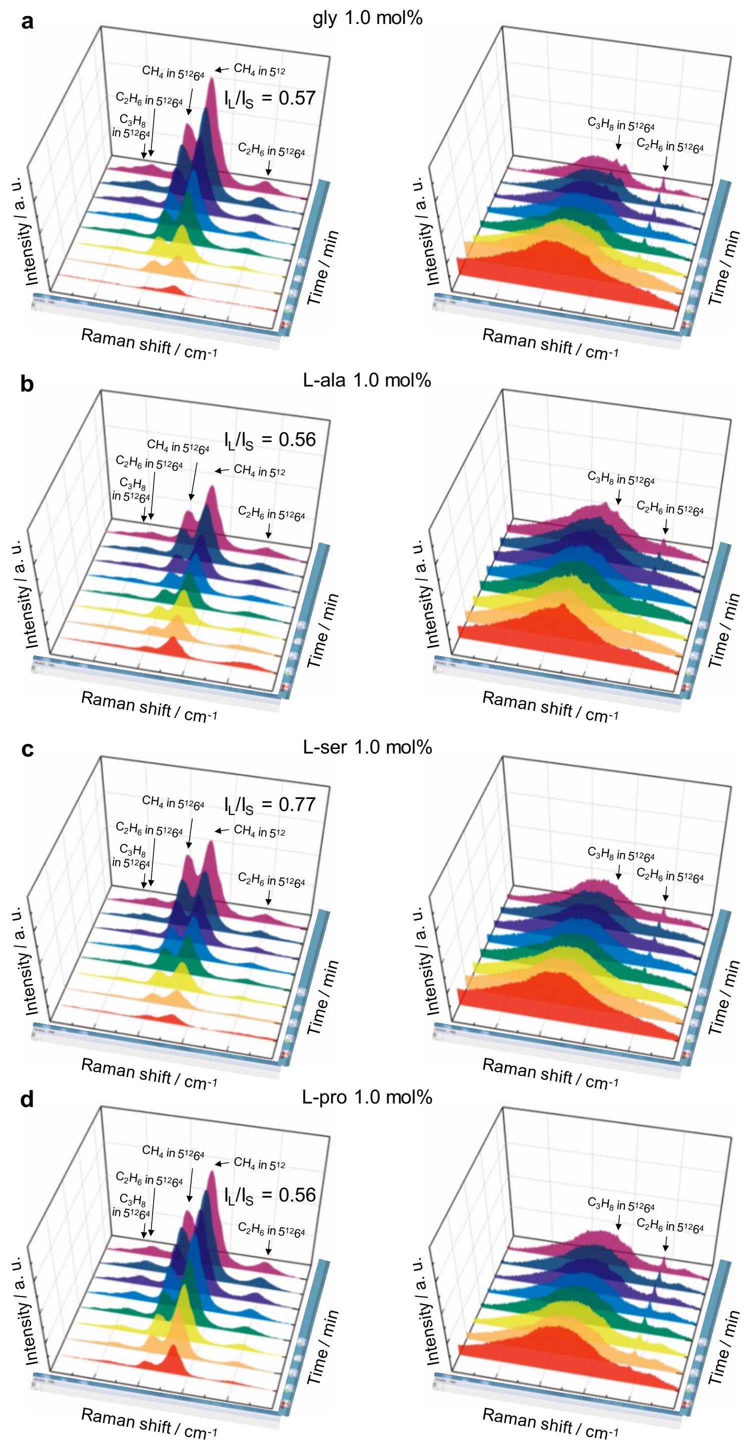

Figure S3. In situ Raman spectra of NG hydrates at 274.15 K in the presence of (a) glycine, (b) L-alanine, (c) L-serine, and (d) L-proline for the C-H and C-C stretching regions. I_L/I_S is the ratio of the peak intensities of the large and small hydrate cages.

6. Hydrate phase equilibrium data

Table S5. Data for CH_4 hydrate phase equilibria in the presence of amino acids.

Pure water		gly 0.5 mol%		gly 1.3 mol%		gly 2.2 mol%		gly 3.0 mol%	
T (K)	P (bar)	T (K)	P (bar)	T (K)	P (bar)	T (K)	P (bar)	T (K)	P (bar)
274.65	29.63	274.45	29.40	273.95	29.53	273.35	29.42	272.85	29.16
278.75	44.48	278.35	44.28	277.65	44.07	277.25	44.16	276.75	43.98
281.55	59.45	281.15	59.28	280.45	59.10	279.95	58.99	279.45	58.78
283.55	74.61	283.25	74.25	282.45	73.95	282.05	74.01	281.40	73.49
285.15	89.96	284.85	89.65	284.30	89.30	283.75	89.23	283.05	88.71

L-ala 0.5 mol%		L-ala 1.3 mol%		L-ala 2.2 mol%		L-ser 1.3 mol%		L-ser 3.0 mol%	
T (K)	P (bar)	T (K)	P (bar)	T (K)	P (bar)	T (K)	P (bar)	T (K)	P (bar)
274.25	29.47	273.95	29.53	273.05	29.32	273.75	29.38	272.65	29.37
278.25	44.13	277.65	44.07	276.95	44.03	277.45	44.23	276.50	44.00
281.05	59.19	280.35	59.08	279.85	59.01	280.25	59.17	279.15	58.85
283.25	74.33	282.35	73.61	281.92	73.93	282.25	74.25	281.20	73.81
284.85	89.52	284.15	89.28	283.58	89.14	284.05	89.40	282.85	88.89

L-pro 1.3 mol%		L-pro 3.0 mol%		L-pro 6.0 mol%		L-pro 9.0 mol%	
T (K)	P (bar)	T (K)	P (bar)	T (K)	P (bar)	T (K)	P (bar)
273.65	29.41	272.30	29.29	268.40	28.87	264.90	28.39
277.35	44.21	276.15	44.05	272.49	43.33	268.10	42.43
280.05	59.06	278.85	58.74	275.05	57.77	270.65	56.62
282.25	74.16	280.95	73.78	277.00	72.31	272.65	70.74
283.85	89.34	282.50	88.68	278.65	86.98	274.00	84.73

Table S6. Data for NG hydrate phase equilibria in the presence of amino acids.

Pure water		gly 0.5 mol%		gly 1.3 mol%		gly 2.2 mol%		gly 3.0 mol%	
T (K)	P (bar)	T (K)	P (bar)	T (K)	P (bar)	T (K)	P (bar)	T (K)	P (bar)
276.30	12.75	276.25	12.48	275.85	12.43	275.45	12.47	274.85	12.45
280.70	19.54	280.45	19.47	280.15	19.52	279.75	19.50	279.15	19.43
283.30	26.56	283.05	26.56	282.65	26.53	282.25	26.43	281.75	26.51
285.25	33.85	285.05	33.71	284.75	33.55	284.35	33.55	283.85	33.55
287.05	40.94	286.75	40.86	286.45	41.03	285.95	40.88	285.35	40.70

L-ala 0.5 mol%		L-ala 1.3 mol%		L-ala 2.2 mol%		L-ser 1.3 mol%		L-ser 3.0 mol%	
T (K)	P (bar)	T (K)	P (bar)	T (K)	P (bar)	T (K)	P (bar)	T (K)	P (bar)
276.15	12.51	275.75	12.45	275.15	12.37	275.45	12.40	274.05	12.36
280.15	19.48	279.95	19.50	279.45	19.50	279.75	19.49	278.35	19.38
282.95	26.55	282.55	26.55	282.05	26.55	282.55	26.55	281.45	26.43
284.85	33.71	284.55	33.57	284.15	33.59	284.45	33.73	283.45	33.50
286.65	41.02	286.35	41.06	285.75	40.86	286.15	40.67	284.75	40.52

L-pro 1.3 mol%		L-pro 3.0 mol%		L-pro 6.0 mol%		L-pro 9.0 mol%	
T (K)	P (bar)	T (K)	P (bar)	T (K)	P (bar)	T (K)	P (bar)
274.85	12.41	273.65	12.34	270.75	12.35	267.65	12.06
279.35	19.46	277.65	19.30	274.50	19.03	271.25	18.91
282.05	26.46	280.45	26.31	277.10	25.95	274.00	25.68
283.95	33.60	282.45	33.39	279.20	32.92	275.70	32.57
285.45	40.66	283.75	40.55	280.65	39.95	276.75	39.32

7. Regression of hydrate phase equilibrium data with an Antoine-like equation

Antoine-like equation: $\ln P\ (\text{bar}) = a + b/T\ (\text{K})$

Table S7. Coefficients of the regression equation fitted to CH_4 hydrate phase equilibria.

System	Concentration (mol%)	a	b
Pure water	-	34.4404	-8540.8199
gly	0.5	34.3362	-8502.7111
	1.3	33.9824	-8384.0658
	2.2	34.0895	-8399.8548
	3.0	34.8552	-8598.0141
L-ala	0.5	33.7398	-8333.2723
	1.3	34.4614	-8516.3482
	2.2	33.7328	-8294.9022
L-ser	1.3	34.2065	-8440.1339
	3.0	34.6077	-8520.9912
L-pro	1.3	34.3594	-8479.5797
	3.0	34.4927	-8479.6671
	6.0	34.3103	-8317.8703
	9.0	36.0147	-8654.0903

Table S8. Coefficients of the regression equation fitted to NG hydrate phase equilibria.

System	Concentration (mol%)	a	b
Pure water	-	35.1041	-9011.5565
gly	0.5	35.8276	-9210.4818
	1.3	35.6570	-9151.4956
	2.2	35.8975	-9206.2017
	3.0	35.7538	-9148.0186
L-ala	0.5	35.5808	-9135.0064
	1.3	35.5282	-9110.2807
	2.2	35.5595	-9103.2140
L-ser	1.3	35.2683	-9032.8011
	3.0	35.0677	-8938.4139
L-pro	1.3	36.0779	-9244.6081
	3.0	36.7703	-9388.7323
	6.0	36.2210	-9133.3231
	9.0	39.2628	-9857.5821

8. Quantitative analysis of hydrate phase equilibrium data

The ΔT and ΔP values were calculated by performing quantitative analyses of the hydrate phase equilibrium data.

Table S9. Quantitative analysis of the CH_4 hydrate phase equilibrium data.

gly 0.5 mol%					
T (K)	P (bar)	T_0 (K)	ΔT (K)	P_0 (bar)	ΔP (bar)
274.45	29.40	274.9834	-0.53337	27.67788	1.722119
278.35	44.28	278.6577	-0.30765	42.80508	1.474916
281.15	59.28	281.3355	-0.18553	58.10427	1.175726
283.25	74.25	283.4378	-0.18778	72.78146	1.468540
284.85	89.65	285.2218	-0.37178	86.21378	3.436218
gly 1.3 mol%					
T (K)	P (bar)	T_0 (K)	ΔT (K)	P_0 (bar)	ΔP (bar)
273.95	29.53	275.0224	-1.07244	26.14964	3.380363
277.65	44.07	278.6144	-0.96444	39.61858	4.451418
280.45	59.10	281.3073	-0.85735	53.86149	5.238513
282.45	73.95	283.3997	-0.94970	66.82367	7.126335
284.3	89.30	285.1845	-0.88453	81.35518	7.944816
gly 2.2 mol%					
T (K)	P (bar)	T_0 (K)	ΔT (K)	P_0 (bar)	ΔP (bar)
273.35	29.42	274.9894	-1.63939	24.42002	4.999984
277.25	44.16	278.6330	-1.38298	37.89874	6.261257
279.95	58.99	281.2901	-1.34009	51.01011	7.979889
282.05	74.01	283.4073	-1.35733	64.01860	9.991403
283.75	89.23	285.1771	-1.42706	76.75313	12.47687
gly 3.0 mol%					
T (K)	P (bar)	T_0 (K)	ΔT (K)	P_0 (bar)	ΔP (bar)
272.85	29.16	274.9108	-2.06082	23.06108	6.098919
276.75	43.98	278.5959	-1.84586	35.84708	8.132919
279.45	58.78	281.2571	-1.80705	48.30028	10.47972
281.40	73.49	283.3410	-1.94104	59.69377	13.79623
283.05	88.71	285.1214	-2.07142	71.24720	17.46280

L-ala 0.5 mol%					
T (K)	P (bar)	T_0 (K)	ΔT (K)	P_0 (bar)	ΔP (bar)
274.25	29.47	275.0044	-0.75442	27.05682	2.413180
278.25	44.13	278.6268	-0.37680	42.33565	1.794352
281.05	59.19	281.3214	-0.27145	57.47962	1.710382
283.25	74.33	283.4479	-0.19791	72.78146	1.548540
284.85	89.52	285.2080	-0.35796	86.21378	3.306218
L-ala 1.3 mol%					
T (K)	P (bar)	T_0 (K)	ΔT (K)	P_0 (bar)	ΔP (bar)
273.95	29.53	275.0224	-1.07244	26.14964	3.380363
277.65	44.07	278.6144	-0.96444	39.61858	4.451418
280.35	59.08	281.3042	-0.95421	53.27956	5.800436
282.35	73.61	283.3564	-1.00637	66.11183	7.498166
284.15	89.28	285.1824	-1.0324	80.07518	9.204824
L-ala 2.2 mol%					
T (K)	P (bar)	T_0 (K)	ΔT (K)	P_0 (bar)	ΔP (bar)
273.05	29.32	274.9592	-1.90925	23.59593	5.724070
276.95	44.03	278.6062	-1.65618	36.65495	7.375046
279.85	59.01	281.2932	-1.44323	50.45703	8.552965
281.92	73.93	283.3972	-1.47716	63.13089	10.79911
283.58	89.14	285.1675	-1.58745	75.38060	13.75940

L-ser 1.3 mol%					
T (K)	P (bar)	T_0 (K)	ΔT (K)	P_0 (bar)	ΔP (bar)
273.75	29.38	274.9773	-1.22734	25.56076	3.819243
277.45	44.23	278.6474	-1.19738	38.74976	5.480244
280.25	59.17	281.3183	-1.06831	52.70354	6.466461
282.25	74.25	283.4378	-1.18778	65.40711	8.842888
284.05	89.40	285.1952	-1.14518	79.23234	10.16766
L-ser 3.0 mol%					
T (K)	P (bar)	T_0 (K)	ΔT (K)	P_0 (bar)	ΔP (bar)
272.65	29.37	274.9743	-2.32433	22.53761	6.832395
276.50	44.00	278.6000	-2.09999	34.86067	9.139334
279.15	58.85	281.2681	-2.11807	46.73961	12.11039
281.20	73.81	283.3819	-2.18188	58.41900	15.39100
282.85	88.89	285.1407	-2.29071	69.74321	19.14679

L-pro 1.3 mol%					
T (K)	P (bar)	T_0 (K)	ΔT (K)	P_0 (bar)	ΔP (bar)
273.65	29.41	274.9864	-1.33638	25.27099	4.139010
277.35	44.21	278.6433	-1.29326	38.32205	5.887953
280.05	59.06	281.3011	-1.25107	51.56887	7.491134
282.25	74.16	283.4264	-1.17637	65.40711	8.752888
283.85	89.34	285.1888	-1.33879	77.57139	11.76861
L-pro 3.0 mol%					
T (K)	P (bar)	T_0 (K)	ΔT (K)	P_0 (bar)	ΔP (bar)
272.30	29.29	274.9502	-2.65018	21.64818	7.641818
276.15	44.05	278.6103	-2.46031	33.52225	10.52775
278.85	58.74	281.2507	-2.40074	45.22616	13.51384
280.95	73.78	283.3781	-2.42806	56.86126	16.91874
282.50	88.68	285.1182	-2.61820	67.18228	21.49772
L-pro 6.0 mol%					
T (K)	P (bar)	T_0 (K)	ΔT (K)	P_0 (bar)	ΔP (bar)
268.40	28.87	274.8224	-6.42240	13.72424	15.14576
272.49	43.33	278.4606	-5.97061	22.12685	21.20315
275.05	57.77	281.0966	-6.04661	29.62206	28.14794
277.00	72.31	283.1890	-6.18896	36.85958	35.45042
278.65	86.98	284.9341	-6.28408	44.24277	42.73723
L-pro 9.0 mol%					
T (K)	P (bar)	T_0 (K)	ΔT (K)	P_0 (bar)	ΔP (bar)
264.90	28.39	274.6742	-9.77422	9.013511	19.37649
268.10	42.43	278.2702	-10.1702	13.24415	29.18585
270.65	56.62	280.9107	-10.2607	17.88038	38.73962
272.65	70.74	282.9830	-10.3330	22.53761	48.20239
274.00	84.73	284.6852	-10.6852	26.29884	58.43116

Table S10. Quantitative analysis of the NG hydrate phase equilibrium data.

gly 0.5 mol%					
T (K)	P (bar)	T_0 (K)	ΔT (K)	P_0 (bar)	ΔP (bar)
276.25	12.48	276.5980	-0.34804	11.97811	0.501888
280.45	19.47	280.4261	0.02388	19.52335	-0.053350
283.05	26.56	283.1624	-0.11240	26.22647	0.333532
285.05	33.71	285.2995	-0.24948	32.79086	0.919140
286.75	40.86	287.0476	-0.29757	39.55031	1.309695
gly 1.3 mol%					
T (K)	P (bar)	T_0 (K)	ΔT (K)	P_0 (bar)	ΔP (bar)
275.85	12.43	276.5640	-0.71396	11.42471	1.005291
280.15	19.52	280.4485	-0.29850	18.86299	0.657008
282.65	26.53	283.1523	-0.50234	25.07105	1.458953
284.75	33.55	285.2565	-0.50651	31.71668	1.833319
286.45	41.03	287.0855	-0.63553	38.26977	2.760229
gly 2.2 mol%					
T (K)	P (bar)	T_0 (K)	ΔT (K)	P_0 (bar)	ΔP (bar)
275.45	12.47	276.5912	-1.14123	10.89538	1.574624
279.75	19.50	280.4396	-0.68956	18.01506	1.484939
282.25	26.43	283.1187	-0.86875	23.96347	2.466533
284.35	33.55	285.2565	-0.90651	30.33566	3.214337
285.95	40.88	287.0520	-1.10204	36.22146	4.658543
gly 3.0 mol%					
T (K)	P (bar)	T_0 (K)	ΔT (K)	P_0 (bar)	ΔP (bar)
274.85	12.45	276.5776	-1.72761	10.14438	2.305621
279.15	19.43	280.4082	-1.25817	16.80994	2.620061
281.75	26.51	283.1456	-1.39563	22.64346	3.866540
283.85	33.55	285.2565	-1.40651	28.68858	4.861419
285.35	40.70	287.0117	-1.66170	33.89904	6.800955

L-ala 0.5 mol%					
T (K)	P (bar)	T_0 (K)	ΔT (K)	P_0 (bar)	ΔP (bar)
276.15	12.51	276.6184	-0.46842	11.83745	0.672550
280.15	19.48	280.4306	-0.28060	18.86299	0.617008
282.95	26.55	283.1590	-0.20905	25.93302	0.616976
284.85	33.71	285.2995	-0.44948	32.07102	1.638977
286.65	41.02	287.0833	-0.43331	39.11907	1.900932
L-ala 1.3 mol%					
T (K)	P (bar)	T_0 (K)	ΔT (K)	P_0 (bar)	ΔP (bar)
275.75	12.45	276.5776	-0.82761	11.29016	1.159842
279.95	19.50	280.4396	-0.48956	18.43445	1.065545
282.55	26.55	283.1590	-0.60905	24.78974	1.760260
284.55	33.57	285.2619	-0.71189	31.01897	2.551027
286.35	41.06	287.0922	-0.74222	37.85163	3.208374
L-ala 2.2 mol%					
T (K)	P (bar)	T_0 (K)	ΔT (K)	P_0 (bar)	ΔP (bar)
275.15	12.37	276.5229	-1.37290	10.51358	1.856417
279.45	19.50	280.4396	-0.98956	17.40272	2.097282
282.05	26.55	283.1590	-1.10905	23.42704	3.122962
284.15	33.59	285.2673	-1.11727	29.66648	3.923524
285.75	40.86	287.0476	-1.29757	35.43125	5.428746

L-ser 1.3 mol%					
T (K)	P (bar)	T_0 (K)	ΔT (K)	P_0 (bar)	ΔP (bar)
275.45	12.40	276.5435	-1.09345	10.89538	1.504624
279.75	19.49	280.4351	-0.68508	18.01506	1.474939
282.55	26.55	283.1590	-0.60905	24.78974	1.760260
284.45	33.73	285.3048	-0.85484	30.67554	3.054465
286.15	40.67	287.0050	-0.85496	37.02814	3.641859
L-ser 3.0 mol%					
T (K)	P (bar)	T_0 (K)	ΔT (K)	P_0 (bar)	ΔP (bar)
274.05	12.36	276.5160	-2.46603	9.218462	3.141538
278.35	19.38	280.3857	-2.03569	15.32046	4.059544
281.45	26.43	283.1187	-1.66875	21.88450	4.545496
283.45	33.50	285.2430	-1.79305	27.43165	6.068350
284.75	40.52	286.9712	-2.22119	31.71668	8.803319

L-pro 1.3 mol%					
T (K)	P (bar)	T_0 (K)	ΔT (K)	P_0 (bar)	ΔP (bar)
274.85	12.41	276.5503	-1.70029	10.14438	2.265621
279.35	19.46	280.4216	-1.07164	17.20298	2.257020
282.05	26.46	283.1288	-1.07884	23.42704	3.032962
283.95	33.60	285.2700	-1.31996	29.01114	4.588861
285.45	40.66	287.0027	-1.55271	34.27617	6.383831
L-pro 3.0 mol%					
T (K)	P (bar)	T_0 (K)	ΔT (K)	P_0 (bar)	ΔP (bar)
273.65	12.34	276.5023	-2.85229	8.785851	3.554149
277.65	19.30	280.3496	-2.69961	14.11964	5.180362
280.45	26.31	283.0783	-2.62827	19.52335	6.786651
282.45	33.39	285.2134	-2.76335	24.51139	8.878606
283.75	40.55	286.9779	-3.22795	28.36939	12.18061
L-pro 6.0 mol%					
T (K)	P (bar)	T_0 (K)	ΔT (K)	P_0 (bar)	ΔP (bar)
270.75	12.35	276.5092	-5.75917	6.174448	6.175552
274.50	19.03	280.2268	-5.72679	9.729034	9.300966
277.10	25.95	282.9558	-5.85581	13.23871	12.71129
279.20	32.92	285.0854	-5.88544	16.90740	16.01260
280.65	39.95	286.8418	-6.19178	19.97556	19.97444
L-pro 9.0 mol%					
T (K)	P (bar)	T_0 (K)	ΔT (K)	P_0 (bar)	ΔP (bar)
267.65	12.06	276.3077	-8.65771	4.199302	7.860698
271.25	18.91	280.1717	-8.92168	6.565127	12.34487
274.00	25.68	282.8629	-8.86292	9.163312	16.51669
275.70	32.57	284.9891	-9.28908	11.22344	21.34656
276.75	39.32	286.6967	-9.94672	12.70527	26.61473

9. Polarized Raman spectra

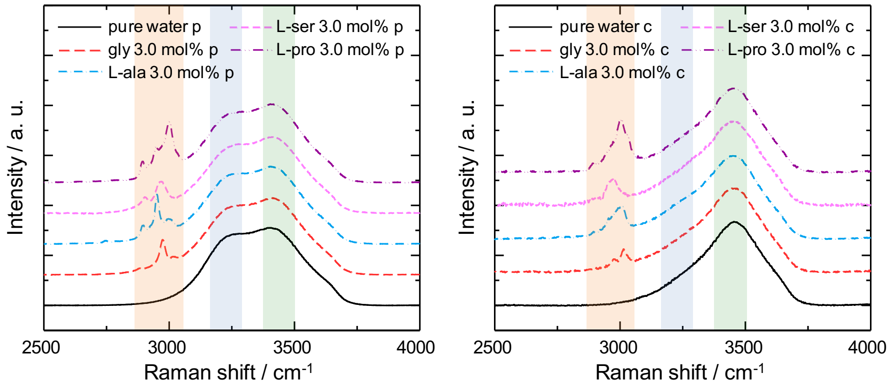

Figure S4. Polarized Raman spectra of the aqueous amino acid solutions for the parallel and cross polarizations. The orange region indicates the unique stretching band of each amino acid[13]. The blue and green regions indicate the collective and non-collective bands of water molecules, respectively.

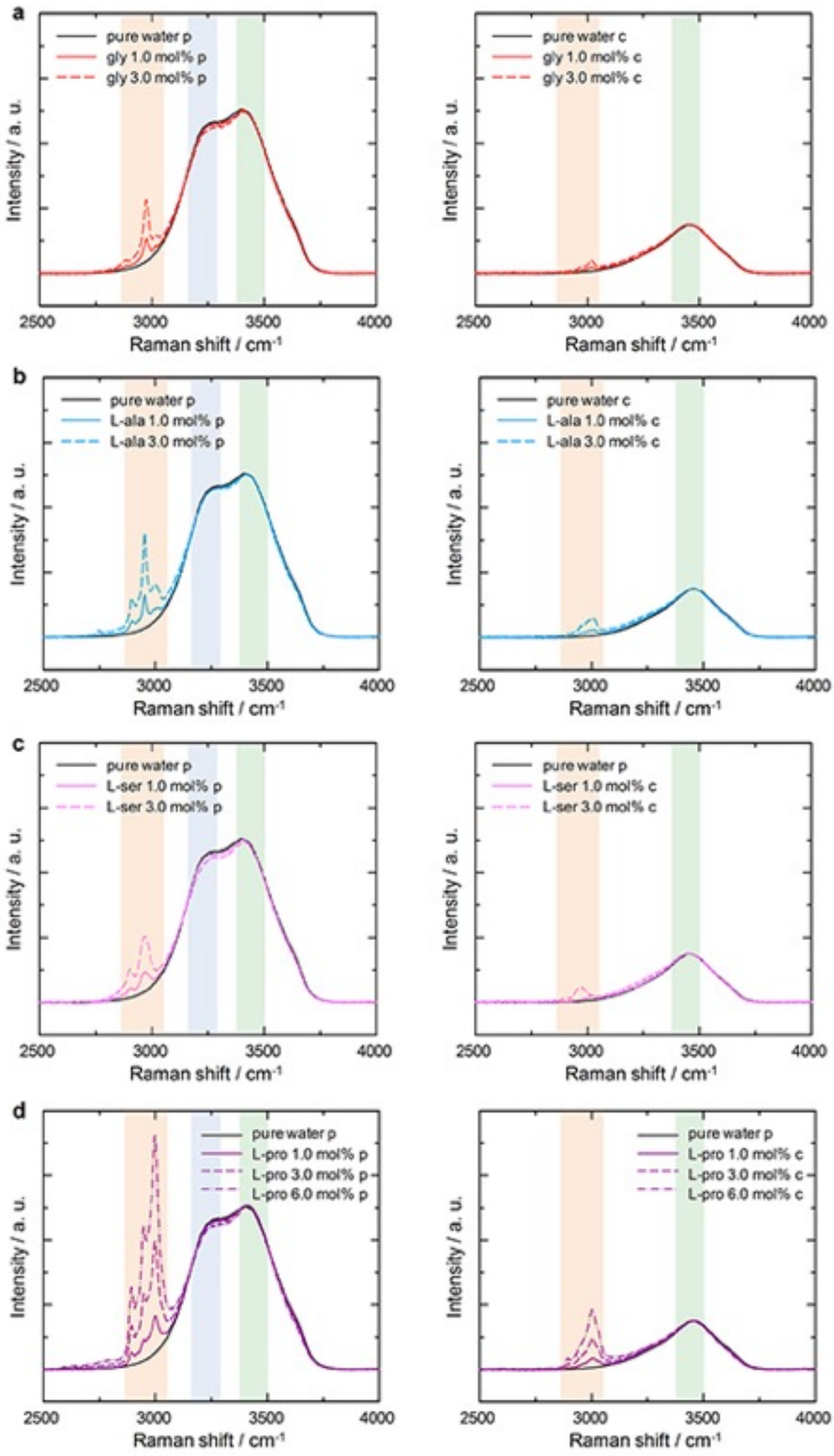

Figure S5. Polarized Raman spectra of aqueous solutions containing (a) glycine, (b) L-alanine, (c) L-serine, and (d) L-proline for the parallel (p) and cross (c) polarizations.

10. Experimental investigation of THF hydrate crystal morphologies

The morphologies of THF hydrate crystals were investigated. Pure THF hydrate crystals are octahedral (Fig. S6a) because their (111) faces are the slowest growing planes[1,2]. In the presence of PVP, THF hydrate crystals are planar (Fig. S6b), as is consistent with previous results[1,2]. This planarity is attributed to the preferential adsorption of PVP onto the (111) faces, which interrupts further hydrate growth. Interestingly, it has previously been demonstrated that polyvinyl caprolactam completely inhibits the growth of THF hydrate crystals[14]. This inhibition of hydrate growth by polymeric hydrate inhibitors has been explained with the adsorption inhibition hypothesis[15]. However, the addition of amino acids does not alter the morphologies of THF hydrate crystals (Figs. S6c to S6f), which implies that adsorption phenomena do not explain hydrate inhibition by amino acids. Amino acids readily form hydrogen bonds with water molecules, but their molecular sizes are too small for efficient blocking of hydrate surfaces. The inhibition of hydrate formation by amino acids can be explained by the perturbation of the structure of water.

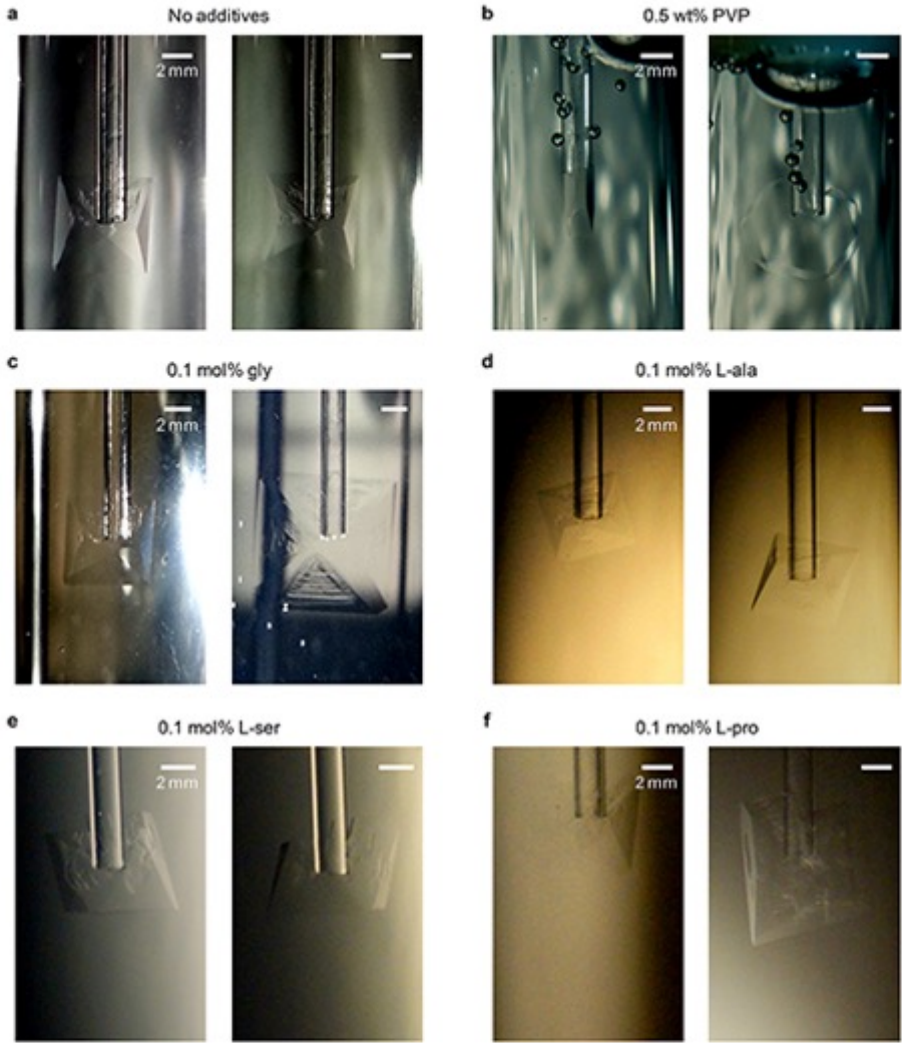

Figure S6. Morphologies of THF hydrate crystals with (a) no additives, (b) PVP, (c) glycine, (d) L-alanine, (e) L-serine, and (f) L-proline.

References

(1) Makogon, T. Y., Larsen, R., Knight, C. A. & Sloan, E. D. Melt growth of tetrahydrofuran clathrate hydrate and its inhibition: method and first results. *J. Cryst. Growth* **179**, 258–262 (1997).

(2) Larsen, R., Knight, C. A. & Sloan, E. D. Clathrate hydrate growth and inhibition. *Fluid Phase Equilibr.* **150–151**, 353–360 (1998).

(3) Ballard, A. L. & Sloan, E. D. The next generation of hydrate prediction I. Hydrate standard states and incorporation of spectroscopy. *Fluid Phase Equilibr.* **194–197**, 371–383 (2002).

(4) Dunn, M. S., Ross, F. J. & Read, L. S. The solubility of the amino acids in water. *J. Biol. Chem.* **103**, 579-595 (1933).

(5) Fasman, G. D. *Handbook of Biochemistry and Molecular Biology* (CRC Press, Cleveland, OH, 1976).

(6) Amend, J. P. & Helgeson, H. C. Solubilities of the common L-α-amino acids as a function of temperature and solution pH. *Pure Appl. Chem.* **69**, 935–942 (1997).

(7) Kyte, J. & Doolittle, R. F. A simple method for displaying the hydropathic character of a protein. *J. Mol. Biol.* **157**, 105–132 (1982).

(8) Bruice, P. Y. *Organic Chemistry* (Pearson Education, Upper Saddle River, NJ, 2007).

(9) Kiss, T., Sovago, I. & Gergely, A. Critical Survey of stability constants of complexes of glycine. *Pure Appl. Chem.* **63**, 597–638 (1991).

(10) Smith, P. K., Taylor, A. C. & Smith, E. R. B. Thermodynamic properties of solutions of amino acids and related substances. III. The ionization of aliphatic amino acids in aqueous solution from one to fifty degrees. *J. Biol. Chem.* **122**, 109–123 (1937).

(11)Sa, J.-H., Kwak, G.-H., Lee, B. R., Ahn, D. & Lee, K.-H. Abnormal incorporation of amino acids into the gas hydrate crystal lattice. *Phys. Chem. Chem. Phys.* **16**, 26730–26734 (2014).

(12)Rodríguez-Carvajal, J. Recent advances in magnetic structure determination by neutron powder diffraction. *Physica B* **192**, 55–69 (1993).

(13)Sa, J.-H., Kwak, G.-H., Han, K., Ahn, D. & Lee, K.-H. Gas hydrate inhibition by perturbation of liquid water structure. *Sci. Rep.* **5**, 11526 (2015).

(14)Carver, T. J., Drew, M. G. B. & Rodger, P. M., Inhibition of crystal growth in methane hydrate. *J. Chem. Soc. Faraday Trans.* **91**, 3449–3460 (1995).

(15)Anderson, B. J., Tester, J. W., Borghi, G. P. & Trout, B. L. Properties of inhibitors of methane hydrate formation via molecular dynamics simulations. *J. Am. Chem. Soc.* **127**, 17852–17862 (2005).
